# Repeated SARS-CoV-2 vaccination in cancer patients treated with immune checkpoint inhibitors: induction of high-avidity anti-RBD neutralizing antibodies

**DOI:** 10.1007/s10147-023-02295-0

**Published:** 2023-01-23

**Authors:** Teresita Caruso, Francesca Salani, Silvia Catanese, Federico Pratesi, Chiara Mercinelli, Giuseppe Motta, Virginia Genovesi, Adele Bonato, Galimberti Sara, Gianluca Masi, Paola Migliorini

**Affiliations:** 1 Clinical Immunology and Allergy Unit, Department of Clinical and Experimental Medicine, University of Pisa, Via Roma, 67, 56126 Pisa, Italy; 2 Oncology Unit, Azienda Ospedaliera Universitaria Pisana, Pisa, Italy; 3 Sant’Anna School of Advanced Studies, Pisa, Italy; 4 Department of Translational Medicine and New Technologies for Medicine and Surgery, University of Pisa, Pisa, Italy; 5 Hematology Unit, Department of Clinical and Experimental Medicine, University of Pisa, Pisa, Italy

**Keywords:** SARS-CoV-2, MRNA vaccine, Anti-Spike antibodies, Neutralizing antibodies, Antibody avidity, Immune Checkpoint inhibitors

## Abstract

**Background:**

Cancer patients are more vulnerable to COVID-19 and are thus given high priority in vaccination campaigns. In solid cancer patients treated with checkpoint inhibitors, we evaluated the amount of anti-RBD and neutralizing antibodies and antibody avidity after two or three doses of the vaccine.

**Methods:**

Thirty-eight solid cancer patients, 15 untreated hematological patients and 21 healthy subjects were enrolled in the study. Blood was collected before the first dose (T0), 21 days after the second (T2) and in 18 solid cancer patients also 15 days after the third dose of vaccine (T3). IgG, IgM and IgA anti-RBD antibodies were detected by ELISA. Neutralizing antibodies were measured testing the inhibition of RBD binding to ACE2. Antibody avidity was evaluated in 18 patients by a urea avidity ELISA.

**Results:**

IgG anti-RBD antibodies were produced in 65.8% of the cancer patients at T2, and in 60% of hematological patients at levels lower than healthy controls. IgM and IgA anti-RBD antibodies were also produced in 5.3% and 21% cancer patients, respectively. At T3, a significant increase in anti-RBD IgG levels was observed. Neutralizing antibodies were produced in 68.4% of cancer patients as compared with 93% of untreated hematological patients and 100% of controls, at titers lower than in healthy subjects. At T3, neutralizing antibodies and avidity of IgG anti-RBD increased; 6/18 patients negative at T2 developed neutralizing antibodies at T3.

**Conclusion:**

The data indicate that in cancer patients mRNA vaccine induces high avidity anti-RBD antibodies and neutralizing antibodies that increase after the third dose. The process of induction and selection of high-affinity antibodies is apparently unaffected by the treatment with anti-PD-1 or anti-PD-L1 antibodies.

## Introduction

Cancer patients have a higher risk of contracting COVID-19 and manifesting a severe form of the disease, with a higher fatality rate [1]. So far, vaccination represents the best strategy to fight the disease, as shown by the results obtained with the widespread administration to millions of people of DNA- or mRNA-based SARS-CoV-2 vaccines. Cancer patients, even if not included in any vaccine registration trials were given high priority in vaccination campaigns.

Several studies have investigated the immune response to SARS-CoV-2 vaccines in cancer patients at different time points after vaccination, obtaining rather homogeneous results.

Among patients with solid tumors that were vaccinated with mRNA vaccine, 75–90% produced anti-spike IgG antibodies as compared with 95–100% of the controls; antibody titers were lower in patients than in controls [2–7]. A recent meta-analysis reported seroconversion in 51% of cancer patients after one dose of vaccine and in 73% after two doses [8].

Antibody activity was also explored, testing the amount of neutralizing antibodies induced by vaccination by means of traditional or surrogate neutralization assays.

The ability of sera to inhibit in vitro the infection of a cell line by a primary isolate of SARS-CoV-2 has been employed to measure neutralizing antibodies in a cohort of cancer patients [9]. Protective titers of neutralizing antibodies were achieved by 86% normal subjects, 86% patients undergoing targeted/hormonal therapy, 53% immunotherapy and 45% chemotherapy [9].

Functional humoral responses induced by vaccination were tested by a live-virus neutralization assays against wild-type virus and variants [10]. After 2 doses of mRNA vaccine, 98% cancer patients and 100% healthy controls developed neutralizing antibodies, with lower titers against the variants analyzed.

Using a secreted *Gaussia*-luciferase SARS-CoV-2-pseudotyped lentivirus neutralization assay, Zeng et al. [11] report that 31% of lung cancer patients and 30% of breast cancer did not develop neutralizing antibodies after vaccination.

The results obtained by traditional neutralization assays, as the ones described above, are highly correlated with the inhibition of interaction between Receptor Binding Domain and Angiotensin Converting Enzyme2 receptor (RBD-ACE2). Thus, assays based on antibody-mediated blockage of ACE2-RBD interaction have been proposed as a SARS-CoV-2 surrogate virus neutralization tests.

Evaluating the inhibition of RBD-ACE2 interaction, Terpos et al. [12] reported that three weeks after the first dose 25% cancer patients vs 65,7% controls developed inhibitory antibodies; the median inhibition titer was lower in patients (p < 0.001).

All the studies show that an increase in frequency of antibody production and a higher titer of antibodies is achieved with the second dose of vaccine, and recent data indicate a further increase with the third dose [13]. It is known that repeated antigen stimulation induces the production of antibodies with increasing affinity for pathogens [14], but this aspect has not been investigated in cancer patients yet.

The aim of the present study is to evaluate quantitative and qualitative aspects of the immune response elicited by mRNA vaccine in cancer patients treated with checkpoint inhibitors, measuring the amount of anti-RBD and neutralizing antibodies and the avidity of anti-RBD antibodies after two or three doses of the vaccine.

## Patients and methods

Thirty-eight patients (22 males, 16 females, age 45–84, mean age 69 years) with different cancers receiving immune check-point inhibitors (ICI) at the Oncology Unit of Pisa University Hospital, and eligible for SARS CoV-2 vaccination were recruited for the study. The primary cancer site included lung (12), bladder (9), kidney (5), gastrointestinal (5), melanoma (3), Merkel cell (2), larynx (1), hepatocellular carcinoma (1).

Fifteen untreated hematological patients (mean age ±SD = 68 ± 8; M/F = 10/5) affected by chronic lymphocytic leukemia and myeloproliferative neoplasm followed at the Hematology Unit of the University of Pisa and eligible for SARS-CoV-2 vaccination were recruited.

Twenty-one health care workers (HCW), vaccinated with mRNA BNT126b2, served as control group (mean age ± SD = 46.8 ± 12.9; M/F = 5/16).

Whole blood was collected before the first dose (T0) and 21 days after the second (T2). For 18 patients, blood was collected also 15 days after the third dose of vaccine (T3). Sera were collected and kept frozen at − 60 °C until use.

No one among patients or controls had contracted SARS-CoV-2 infection before recruitment in the study.

The study was approved by the local Ethical Committee (Approval N° 17522) and patients signed an informed consent the day of enrolment.

### Anti-RBD antibody titers

Antibodies were measured by solid phase assay, on plates coated with recombinant Receptor Binding Domain (RBD: SARS-CoV-2 Spike protein aa_319–541_), as previously described [15]. IgG, IgM and IgA anti-RBD antibodies were detected.

### Analysis of neutralizing antibodies

To detect neutralizing antibodies, the kit SPIA (Spike Protein Inhibition Assay, DiaMetra, Perugia, Italy) was employed according to manufacturer’s instructions. In this assay, patient’s antibodies compete with peroxidase-conjugated ACE2 for the binding to viral RBD coated on the solid phase.

Inhibition value was calculated using this formula:

$$\% \;{\text{inhibition}} = \left[ {1 - \left( {{\text{Absorbance}}_{{{\text{Sample}}}} } \right)/\left( {{\text{Absorbance}}_{{{\text{Calibrator}}}} } \right)} \right] \times 100.$$

### Avidity assay

Antibody avidity was evaluated in a subgroup of 18 patients, after the second and third dose of vaccine, by means of an Avidity ELISA, employing different concentrations of Urea as chaotropic reagent. The Avidity Index (AI) was calculated as the extrapolated urea concentration that displaces 50% of serum binding with respect to the control wells using the approach previously described [15]. The area under the curve (AUC) obtained plotting on the X-axis the urea concentrations and on the Y-axis the corresponding percentage of binding with respect with the sample not treated with Urea (considered as 100% of binding) was used to compare the avidity of anti-RBD antibodies after the second (T2) and the third dose (T3) of vaccine.

### Statistical analysis

Statistical analysis was performed using IBM-SPSS^®^ Statistics, and GraphPad Prism statistical packages. Antibody levels at different time points were compared by Kruskal–Wallis and one-way ANOVA with Bonferroni correction for multiple comparisons. Results of anti-RBD Ig were expressed as odds ratio (OR) of a positive internal control set at 1.0. Cut-off values have been set at the 97.5th percentile of the normal healthy subjects (NHS) evaluated before vaccination. *p* < 0.05 was considered as significant.

## Results

### Quantitative analysis of anti-RBD antibodies in vaccinated cancer patients

Thirty-eight cancer patients were enrolled in the study. At the time of vaccination, 30 patients were treated with anti PD-1 monoclonal antibodies and 8 with antiPD-L1 antibodies. Previous therapies included chemotherapy in 22, chemotherapy associated with radiotherapy in 10, radiotherapy in 3.

**Fig. 1:**
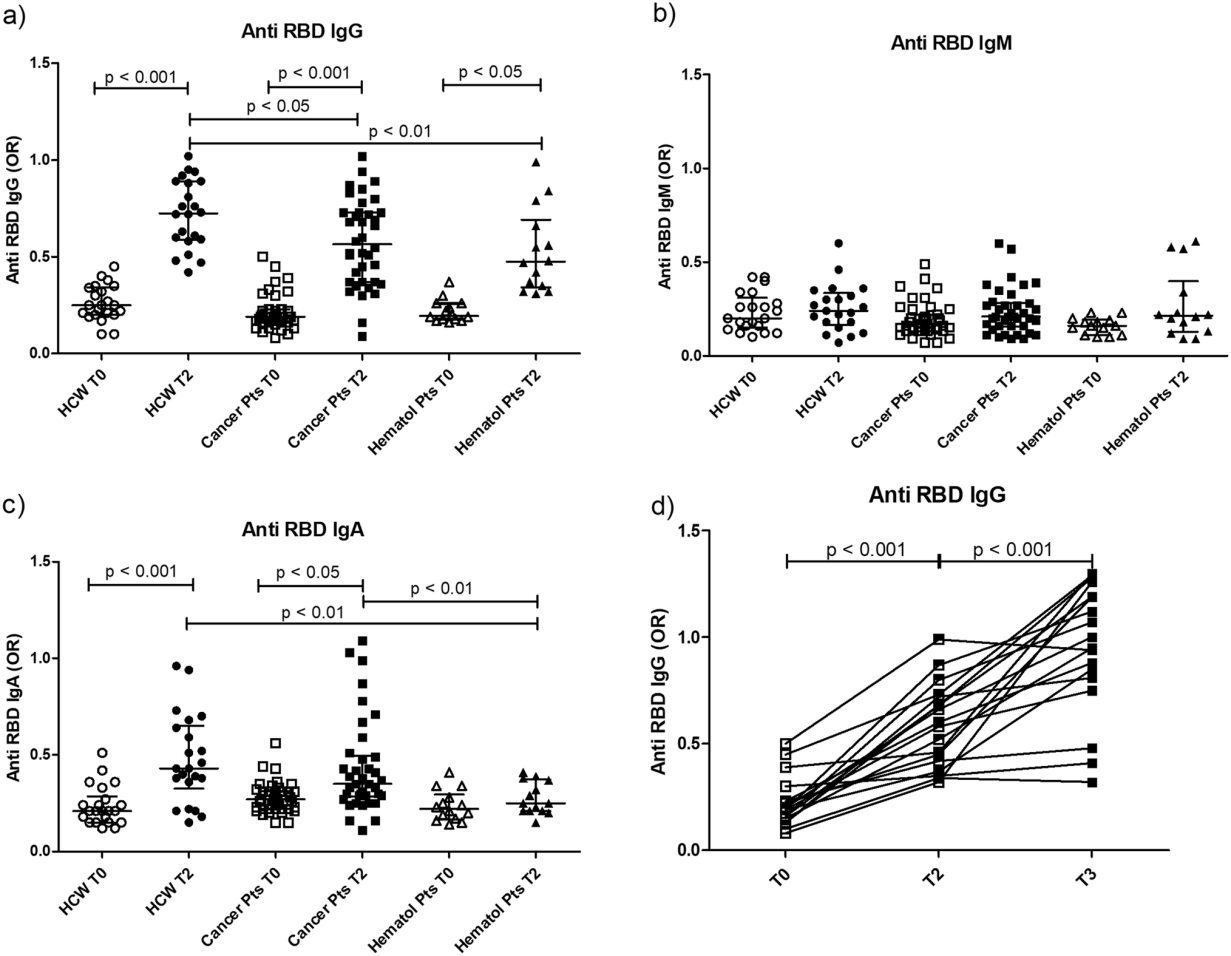
Distribution of anti-RBD immunoglobulins. Distribution of IgG (**a**), IgM (**b**) and IgA (**c**) anti-RBD in solid cancer patients as compared with untreated hematological patients and health care workers (HCW), before the first dose (T0) and after the second (T2). Levels of IgG anti-RBD antibodies in 18 patients that received a third dose before the first dose (T0), after the second (T2) and after the third (T3) (**d**). Results are represented as odds ratio of a positive internal control (OR). *p* < 0.05 was considered as significant

IgG anti-RBD antibodies were produced in 25/38 (65.8%) of the solid cancer patients after the second dose (Fig. 1a) as compared to 60% of the untreated hematological patients and to 100% of healthy subjects. Mean antibody levels, however, were lower than in healthy vaccinated controls (*p* < 0.05).

IgM and IgA anti-RBD antibodies were also produced in 2/38 (5.3%) and 8/38 (21%) solid cancer patients, respectively (Fig. 1b, c); mean IgM and IgA levels did not show any differences with respect to HCW. In hematological patients, after second dose, IgA anti-RBD are produced at very low levels (Fig. 1c).

In 18 cancer patients that were re-evaluated after the third dose (T3), an increase in anti RBD IgG levels was observed (*p* < 0.001, Fig. 1d).

### Qualitative analysis of anti-RBD antibodies in vaccinated cancer patients

Qualitative aspects of the immune response induced by the vaccine in cancer patients were also investigated analyzing the neutralizing ability and the avidity of antibodies.

To evaluate neutralizing antibodies, we tested the ability of sera to inhibit the interaction of SARS-CoV-2 RBD with the human host receptor angiotensin-converting enzyme 2 (ACE2).

**Fig. 2:**
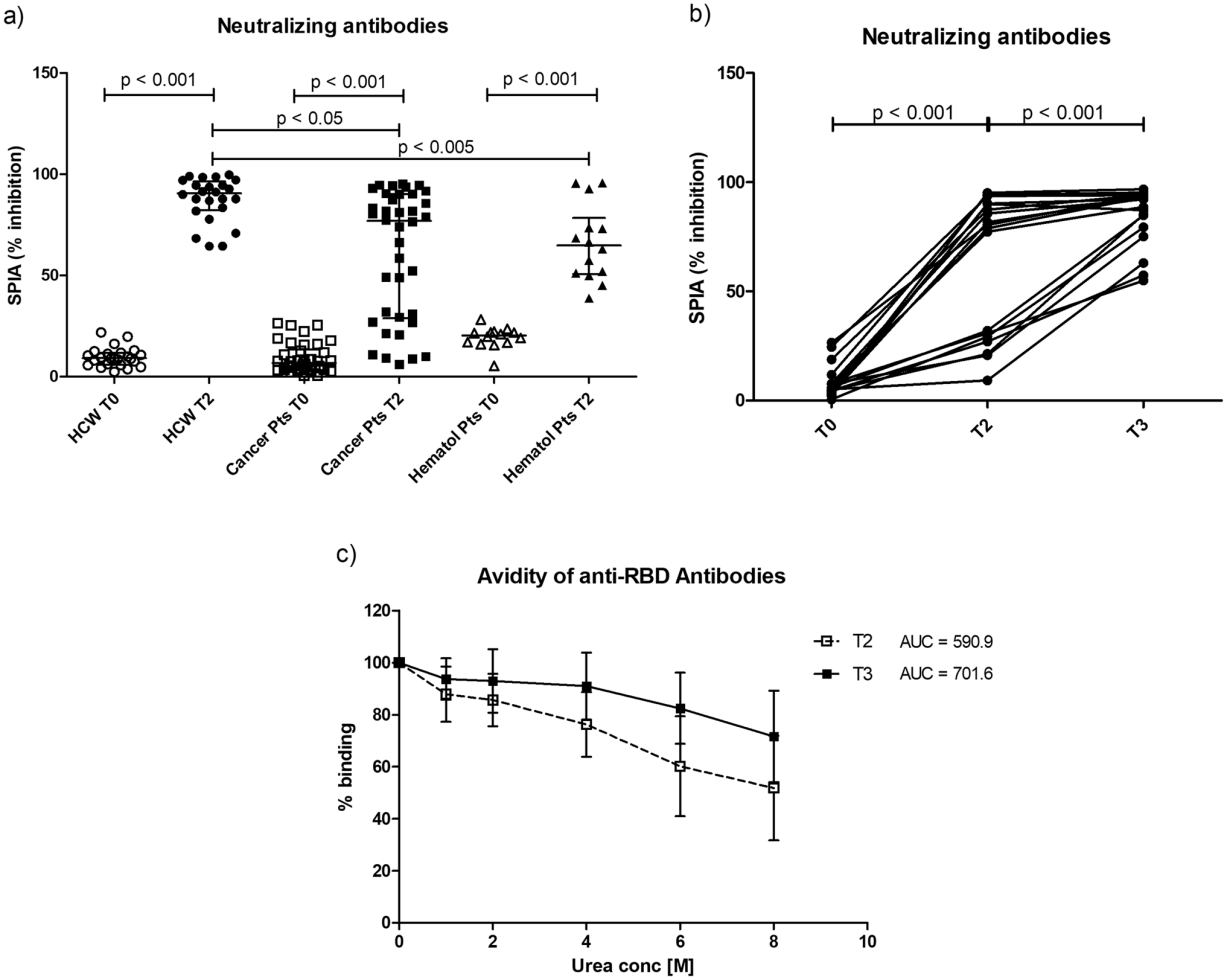
Neutralizing ability and avidity of anti-RBD antibodies. **a** The distribution of immunoglobulin inhibitory activity measured by SPIA kit before the first dose of vaccine (T0) and after the second (T2) in solid cancer patients, hematological subjects and health care workers (HCW). Levels of neutralizing antibodies in 18 solid cancer patients that received a third dose before the first dose (T0), after the second (T2) and after the third dose (T3) (**b**). Results are expressed as the percentage of inhibition of the binding of labeled ACE2 receptor to RBD coated plates. *p* < 0.05 was considered as significant. **c** The avidity of anti RBD IgG from vaccinated solid cancer patients after the second (T2) and after the third dose (T3). For the different urea concentrations, mean binding values and standard deviations obtained in each patient group are represented

Neutralizing antibodies were produced in 26/38 (68.4%) of solid cancer patients as compared with 93% of untreated hematological subjects and 100% of controls. Mean antibody titers were in both patients cohorts lower than in healthy subjects (*p* < 0.05—Fig. 2a). However, the third dose induced an increase in the percentage of neutralizing antibodies (*p* < 0.001) and more interestingly, 6/18 patients negative after the second dose developed neutralizing antibodies after the third (Fig. 2b).

Antibody avidity at T2 and T3 was evaluated in the subgroup of 18 solid cancer subjects who received booster dose, using a chaotropic ELISA. As shown in Fig. 2c, Anti-RBD IgG avidity after the booster dose is higher than avidity after second dose (AUC_T3_ = 701.6 vs AUC_T2_ = 590.9). Hematological patients developed, after the second dose, antibodies with an avidity comparable to the one displayed by solid cancer patients (AUC = 603.4, data not shown).

### Clinical and therapy correlations.

The production of anti-RBD antibodies or neutralizing antibodies was not different in patients treated with anti PD-1 or anti PD-L1. Similarly, previous treatment with chemotherapy or radiotherapy did not influence antibody titer. An inverse correlation was instead observed between neutralizing antibodies and leukocyte or neutrophils count.

## Discussion

The data obtained in the present study confirm that mRNA vaccine is effective in most cancer patients: in fact, high-avidity anti-RBD antibodies and neutralizing antibodies are elicited in solid cancer and in hematological patients. Moreover, repeated vaccine doses induce an increase in titer and avidity of anti-RBD antibodies and a higher amount of neutralizing antibodies.

The percentage of responders and the titer of IgG anti-RBD obtained in our cohort of patients are comparable to what has been previously reported [2–7].

In the studied patients, ongoing or past therapies do not affect the amount of elicited antibodies and this observation is supported by studies previously conducted in ample cohorts of cancer patients. The VOICE trial compared the immune response induced by mRNA vaccines in cancer patients treated with immunotherapy, chemotherapy or both [16]. Most patients achieved protective levels of anti-RBD antibodies after the second dose: the number of non-responders or low responder was 7%. 16% and 11% in the 3 cohorts. Moreover, half of the non-responders developed a specific T cell response, suggesting an efficacy of vaccination despite the low antibody levels [16]. Similarly, solid cancer patients treated with immunotherapy or targeted therapy/hormonal therapy did not differ from controls, while patients undergoing chemotherapy had a significantly lower response [9]. A proportion of these patients under chemotherapy were vaccinated during treatment, at variance with the previous study and with our cohort of patients. Even if no difference was detected between the 2 subcohorts “off cycle” and “on cycle”, proximity to treatment should still be considered an important factor potentially affecting the immune response to vaccine.

The quality of antibodies induced by vaccination has been less frequently studied.

The induction of neutralizing antibodies has been analyzed by different techniques, making difficult a direct comparison of antibody levels obtained in different studies. Even if plaque reduction neutralization tests represent the gold standard for the detection of neutralizing antibodies, a strong correlation between neutralization assays and inhibition of RBD-ACE2 interaction has been observed. Thus, the antibody-mediated blockage of ACE2-spike is presently considered a SARS-CoV-2 surrogate virus neutralization test [17].

By such an assay, we measured potentially protective antibodies of any subclass that inhibit the interaction of RBD with ACE, obtaining a lower titer of neutralizing antibodies in patients vs controls. A reduction in the mean level of neutralizing antibodies in cancer patients under treatment vs normal subjects is a frequent finding [18]. Most patients, however, do produce neutralizing antibodies and such a result mirrors the efficacy of vaccination in preventing the disease as observed in cancer patients [18].

No data are available on the avidity of anti-RBD antibodies induced by vaccine in cancer patients. Our data indicate that the avidity is similar to what observed in healthy controls and, most interestingly, increased by the third vaccine dose.

Repeated antigenic stimulation leads to the selection of B cells that bear a receptor able to bind the antigen with a higher avidity, as a result of clonal selection in germinal centers. Previous studies showed how antibody avidity for pertussis toxin increases over time after infection or vaccination, then declining over time [19]. Analyzing the immune response to a measles-vectored chikungunya vaccine, Tschismarov et al. [20] reported an increase in avidity with the second dose. The data we obtained show a similar trend in SARS CoV 2 vaccination, probably dependent on the expansion of hypermutated memory B cells producing high-avidity antibodies.

The process of induction and selection of high-affinity antibodies is apparently unaffected by the treatment with anti PD-1 or anti PD-L1 antibodies. Such an observation as the ones obtained by other groups [21], coupled with the overall efficacy of vaccination after two and especially three doses and the lack of side effects, strongly supports the current policy of vaccination in cancer patients undergoing immunotherapy.

## Data Availability

All data generated or analysed during this study are included in this published article.
