# Correction to: Repeated SARS-CoV-2 vaccination in cancer patients treated with immune checkpoint inhibitors: induction of high-avidity anti-RBD neutralizing antibodies

**DOI:** 10.1007/s10147-023-02310-4

**Published:** 2023-02-20

**Authors:** Teresita Caruso, Francesca Salani, Silvia Catanese, Federico Pratesi, Chiara Mercinelli, Giuseppe Motta, Virginia Genovesi, Adele Bonato, Galimberti Sara, Gianluca Masi, Paola Migliorini

**Affiliations:** 1 Clinical Immunology and Allergy Unit, Department of Clinical and Experimental Medicine, University of Pisa, Via Roma, 67, 56126 Pisa, Italy; 2 Oncology Unit, Azienda Ospedaliera Universitaria Pisana, Pisa, Italy; 3 Sant’Anna School of Advanced Studies, Pisa, Italy; 4 Department of Translational Medicine and New Technologies for Medicine and Surgery, University of Pisa, Pisa, Italy; 5 Hematology Unit, Department of Clinical and Experimental Medicine, University of Pisa, Pisa, Italy

**Correction to: International Journal of Clinical Oncology** 10.1007/s10147-023-02295-0

In the original publication the given and family names of authors are swapped. The correct names are given in this Correction.

The original publication has been updated.

